# Draft genome sequences of five glacial fungi from Styx Glacier, Antarctica

**DOI:** 10.1128/mra.01175-24

**Published:** 2025-02-27

**Authors:** Ji Seon Kim, Chang Wan Seo, Young Woon Lim

**Affiliations:** 1 School of Biological Sciences, Institute of Biodiversity, Seoul National University 123584 , Seoul, Republic of Korea; University of Guelph, Guelph, Canada

**Keywords:** Antarctica, glacial fungi, whole genome sequence, halotolerance

## Abstract

We generated four high-quality draft genomes of fungi isolated from Styx Glacier, Antarctica. The genome announcement of these fungal strains offers insights into their evolution and adaptation to extreme glacial environments, as well as their potential biotechnological applications.

## ANNOUNCEMENT

Glaciers entrap various ancient elements, such as organic/inorganic particles and gases (1–3), and there have been some reports of entrapped microbes with potential for resuscitation (2, 4, 5). In 2023, we isolated historical fungal strains from glacial cores sampled from the Styx glacier, Antarctica (−73.851667, 163.687000) through collaboration with the Division of Glacier and Earth Sciences of the Korea Polar Research Institute (6). Four strains were selected based on the need for further taxonomic and ecological characterization (e.g., unique traits or understudied taxonomic groups) (see Table 1 for detailed strain information).

**TABLE 1:** Strain information and statistics of the assembled whole-genome sequence for the four glacial fungal strains

Strain no.		Styx10C10M-37	Styx25C50M-22	Styx10C100M-05	Styx25C100M-44
**Assigned taxonomy**		*Leotiomycetes* sp.	*Moesziomyces antarcticus*	*Peroneutypa scoparia*	*Peroneutypa* sp.
**Isolation condition (medium/temp**)		Tryptone soy broth/10°C	Nutrient broth/25°C	Tryptone soy broth/10°C	Potato dextrose broth/25°C
**Genome project information**	GenBank accession no.	ITS: PQ459909 LSU: PQ459912	ITS: PQ459908	ITS: PQ459911	ITS: PQ459910PQ459910
	BioSample ID	SAMN43984000	SAMN43984134	SAMN43984119	SAMN43984135
	Assembly ID	JBIDDL000000000	JBIDDN000000000	JBIDDO000000000	JBIDDM000000000
	BioProject ID	PRJNA1166992	PRJNA1167009	PRJNA1167006	PRJNA1167011
**Illumina data**	SRA accession no.	SRR30908074	SRR30915988	SRR30935107	SRR30935109
	Total bases (bp)	5,829,209,436	5,919,985,200	4,800,517,406	5,339,958,564
	No. of reads	38,604,036	39,205,200	31,791,506	35,363,964
**Nanopore data**	SRA accession no.	SRR30908050	SRR30915987	SRR30935106	SRR30935108
	Total bases (bp)	4,714,916,066	5,531,211,399	5,973,422,600	12,559,060,800
	No. of reads	2,403,038	549,979	252,618	2,186,363
	*N*_50_ (bp)	3,247	22,456	44,087	33,005
**Genome assembly statistics**	Assembler type	NextDenovo	Flye	Flye	NextDenovo
	Contig no.	3	34	15	13
	Genome size (bp)	26,888,994	18,515,233	47,265,203	47,860,354
	Nanopore coverage (×)	102.74	279	96	301.38
	NovaSeq coverage (×)	99	299	91	99
	GC content (%)	47.78	60.78	50.27	49.56
	*N*_50_ no.	2	7	4	4
	*N*_50_ length (bp)	6,884,066	747,440	5,744,238	5,591,736

**Fig 1:**
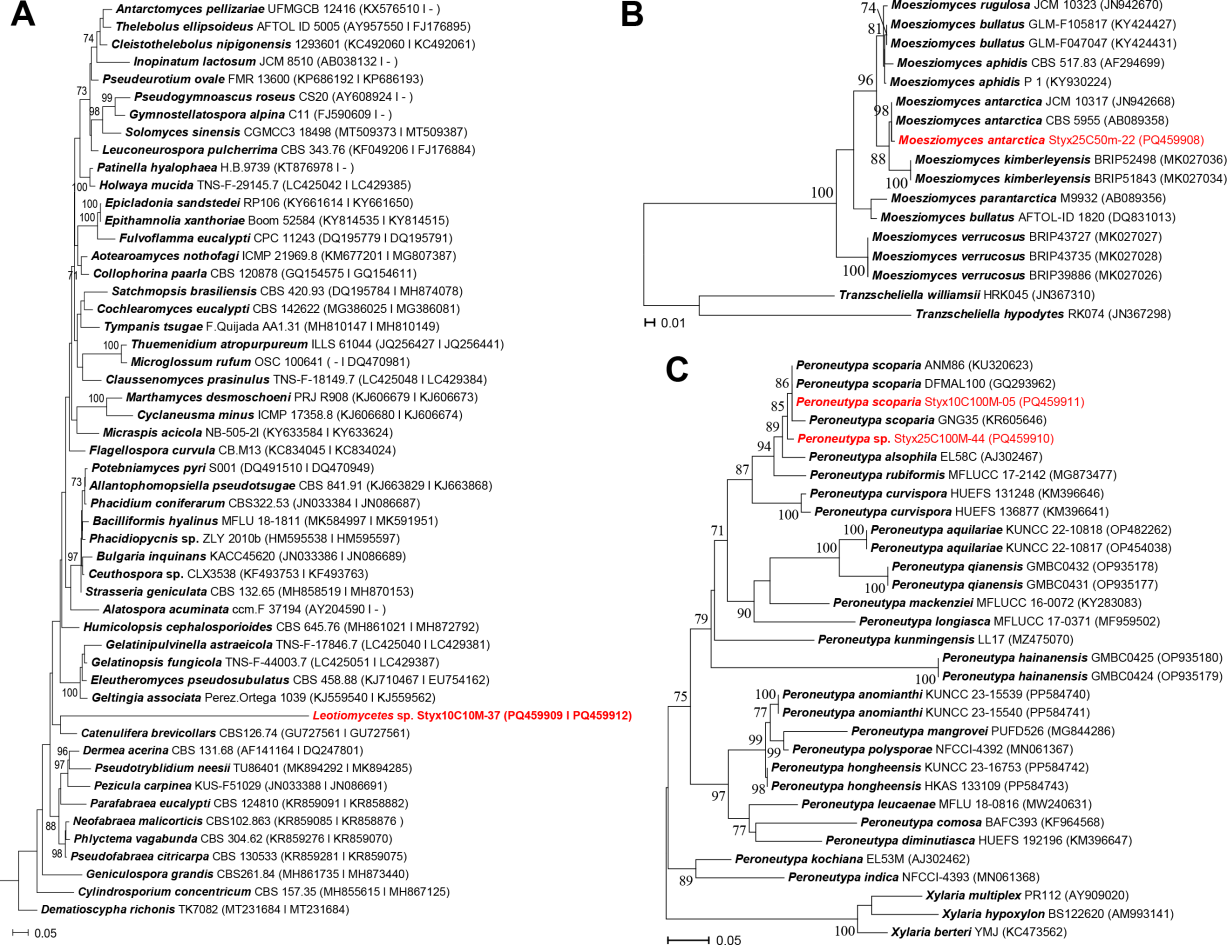
Maximum likelihood phylogenetic trees for the identification of four glacial fungal strains, highlighting those in color. (**A**) Phylogenetic tree of the *Leotiomycetes* lineage based on ITS and LSU for identifying Leotiomycetes sp. (Styx10C10M-37). (**B**) ITS-based phylogenetic tree of the genus *Moesziomyces* for identifying *Moesziomyces antarcticus* (Styx25C50M-22). (**C**) ITS-based phylogenetic tree of the genus *Peroneutypa* lineage for identifying *Peroneutypa scoparia* (Styx10C100M-05) and *Peroneutypa* sp. (Styx25C100M-44). Each branch leaf shows the species name followed by the strain number. GenBank accession numbers for the strains are shown in panel **A** with ITS and LSU in parentheses, and in panels **B** and **C**, only the ITS number is provided.

For taxonomic identification, genomic DNA extraction, PCR amplification, and sequencing were performed following the methods described in previous studies (7). Primer sets ITS1/ITS4 (8) and LR0R/LR5 (9) were used for amplifying ITS and nrLSU regions, respectively, with the same primer sets utilized for both PCR and sequencing. Sequences were manually edited using Geneious Prime version 2024.0.7 (https://www.geneious.com) and then deposited in GenBank (Table 1). Sequences were aligned with reference sequences using MAFFT with the L-INS-i option (10), and a phylogenetic tree was constructed with RAxML employing the GTR + GAMMA model and 1,000 bootstrap replicates (11). Taxonomic identification was finalized based on the phylogenetic trees (Fig. 1).

For genome sequencing, strains for high-molecular-weight (HMW) DNA extraction were grown in 150 mL potato dextrose broth (Difco, USA) at 25°C with shaking at 150 rpm. Mycelia were dehydrated using a vacuum pump (GAST, USA), and HMW DNA was extracted with the Promega Wizard high-molecular-weight (HMW) extraction kit (Madison, WI) or a modified CTAB protocol. Genomic DNA was sequenced using a combination of NovaSeq 6000 and Oxford Nanopore Technologies (ONT) platforms. For long-read sequencing, library preparation and sequencing were conducted at the National Instrumentation Center for Environmental Management, Seoul National University (Republic of Korea). Libraries were constructed using the Ligation Sequencing Kit (SQK-LSK-109; ONT, UK) without shearing. Small DNA fragments were removed with a Circulomics Short Read Eliminator kit, and sequencing was performed on a PromethION 24 with an R9.4.1 flow cell and MinKNOW 21.11.7. Long-read sequence data were filtered and base-called with Guppy version 6.5.7 (https://community.nanoporetech.com). Raw reads were trimmed using Porechop version 0.2.4 (https://github.com/rrwick/Porechop). For short-read polishing, library preparation and sequencing were conducted at Macrogen (Republic of Korea). The library was constructed using the TruSeq Nano DNA kit, followed by sequencing on the Illumina NovaSeq 6000 platform with paired-end 2 × 151 bp reads. Raw reads were trimmed with fastp version 0.23.2 (12) using default settings and assembled using NextDenovo version 2.5.2 (13) or Flye version 2.9.2 (14). The assembled reads were polished with four rounds each of Racon version 1.5.0 (15) and Hapo-G version 1.3.1 (16). Assembly quality and completeness statistics, including *N*_50_ and *L*_50_, were obtained using QUAST version 5.2.0 (17). Tapestry version 1.0.0 was used to visualize sequencing depth uniformity, contiguity, and telomere completeness, with telomere “CTGGTG” (18). Contig selection was performed via Tapestry version 1.0.0. Data from the final assemblies are summarized in Table 1.

## Data Availability

This Whole Genome Sequencing data have been deposited in DDBJ/ENA/GenBank under the accession no. JBIDDL000000000, JBIDDN000000000, JBIDDM000000000, and JBIDDO000000000. The version described in this paper is the first version, JBIDDL010000000, JBIDDN010000000, JBIDDM010000000, and JBIDDO010000000.
