# Correction for Kim et al., “Draft genome sequences of four glacial fungi from Styx Glacier, Antarctica”

**DOI:** 10.1128/mra.00403-25

**Published:** 2025-06-11

**Authors:** Ji Seon Kim, Chang Wan Seo, Young Woon Lim

## ERRATUM

Volume 14, no. 4, e01175-24, 2025, https://doi.org/10.1128/mra.01175-24. The article title should read as given in this erratum.

